# Investigation of Binding Modes and Functional Surface of Scorpion Toxins ANEP to Sodium Channels 1.7

**DOI:** 10.3390/toxins9120387

**Published:** 2017-11-29

**Authors:** Yongbo Song, Zeyu Liu, Qi Zhang, Chunming Li, Wei Jin, Lili Liu, Jianye Zhang, Jinghai Zhang

**Affiliations:** 1 School of Life Sciences and Biopharmaceutical Science, Shenyang Pharmaceutical University, Shenyang 110016, Liaoning, China; liuzeyu.sy@outlook.com (Z.L.); zq15940208604@hotmail.com (Q.Z.); lichunming.sy@outlook.com (C.L.); jinwei0303@hotmail.com (W.J.); liulili.sy@outlook.com (L.L.); zhangjianye94@hotmail.com (J.Z.); 2 School of Medical Devices, Shenyang Pharmaceutical University, Shenyang 110016, Liaoning, China; zhangjinghai@syphu.edu.cn

**Keywords:** *Buthus martensii* Karsch, analgesic activity, ANEP, Na_v_1.7-site 4, molecular dynamics simulations

## Abstract

The depressant β toxin anti-neuroexcitation peptide (ANEP) from the Chinese scorpion *Buthus martensii* Karsch has analgesic activity by interacting with receptor site 4 of the voltage-gated sodium channels (VGSCs). Here, with molecular dynamics simulations, we examined the binding modes between ANEP and the site 4 of mice sodium channel 1.7 (mNa_v_1.7), a subtype of VGSCs related to peripheral pain. Homology modeling, molecular mechanics, and molecular dynamics in the biomembrane environment were adopted. The results suggested that ANEP bound to the resting site 4 mainly by amino acid residues in the β2–β3 loop and the ‘NC’ domains, and the activate site 4 mainly by amino acid residues in the hydrophobic domain of N-groove and residues in the ‘pharmacophore’. Effects analysis of 14 mutants in the predicted functional domains of ANEP on mouse twisting models showed that the analgesic activity of mutants L15 and E24 of the ‘pharmacophore’, W36, T37, W38, and T39 forming the loop between the β2- and β3-strands and N8, V12, C60, and K64 in the NC domain increased distinctly after these residues were substituted for Ala, respectively. The binding modes and the active sites predicted were consistent with available mutagenesis data, and which is meaningful to understand the related mechanisms of ANEP for Na_v_1.7.

## 1. Introduction

Voltage-gated sodium channels (VGSCs) are voltage-dependent transmembrane sodium channels comprised of pore-forming α-subunit associated with up to four different β-subunits (β1–β4). α-subunit consists of four repeat domains (I–IV), each of them containing six trans-membrane segments (S1–S6) that are connected by intra- and extra-cellular loops. The S4 segments serve as voltage sensors to initiate the voltage-dependent activation of sodium channels by moving outward under the influence of the electric field, thereby imposing a conformational change leading to channel activation [1].

VGSCs are key targets for the development of pharmaceutical drugs [2]. Up to date, nine specific voltage-gated sodium channel α-chain subtypes (Na_v_1.1–Na_v_1.9) have been identified and each of them shows tissue specific expression profile. Both human genetic data and transgenic mouse models suggest that individual subtype links to particular types of pain [3]. Recent reports have revealed that nonsense mutations of SCN9A, the encoding gene of Na_v_1.7, are the cause of familial congenital analgesia [4], and dominant mutations of the same gene are responsible for inherited erythromelalgia, a debilitating pain disorder [5]. Mainly distributing in the sympathetic neurons, olfactory epithelium, and dorsal root ganglion sensory neurons, Na_v_1.7 plays a critical role in the development of both acute and chronic peripheral pain that is associated with tissue and nerve injury [6].

Several groups of polypeptide neurotoxins, which can bind to specific receptor sites with high affinity and strongly alter function on the sodium channels, have been studied extensively [7]. According to their modes of action and binding properties to distinct receptor sites, scorpion toxins can be divided into α- and β-classes. β-scorpion toxins are composed of 60–65 amino acid residues that are cross-linked by four disulfide bridges. By binding to receptor site 4, β-scorpion toxins shift the voltage dependence of activation of VGSCs to cause subthreshold opening of channels and reduce the peak current amplitude [8]. The interaction of this toxin with its specific receptor site is dependent on the activated conformational state of the toxin receptor site. The proposed mechanism of β-toxins on sodium channels involves the binding to the S3–S4 loop at the extracellular end of the IIS4 segment in activated sodium channels, which further traps and stabilizes this segment in its outward activated position, hence enhances channel activation in response to the subsequent depolarization and thus causes the negative shift in the voltage dependence of activation. 

The typical effects of β-scorpion toxins are exemplified by the pharmacological activities of Css4 (anti-mammal), BmKITc (anti-insect depressant), Bj-xtrIT (anti-insect excitatory), and Tsγ (both anti-mammal and anti-insect) [9]. Molecular dissection of Bj-xtrIT has elucidated its functional surface composed of two distinct domains: a putative hot spot and another motif associated with the C-tail [10]. Thorough mutagenesis of Css4 suggested that, similar to its anti-insect homolog, this anti-mammalian β-toxin also bound to its specific receptor site via ‘pharmacophore’ comprised of a cluster of conserved residues in the putative hot spot [11]. Meanwhile, a hydrophobic cluster, mainly in the loop connecting β2 and β3 strands of VGSCs seemed to determine the specificity for mammalian sodium channels. In addition, a negatively charged residue (Glu15) was shown to be involved in voltage sensor trapping [12].

The venom of the Asian scorpion *Buthus martensii* Karsch is a very important analgesic in traditional Chinese medicine and is widely used to treat diverse types of pains, such as migraines, rheumatic pain, and cancer pain. Some analgesic peptides have been isolated from *Buthus martensii* Karsch, such as BmK IT2, BmK IT4, BmK AS, BmK AS-1, BmK I1, BmK I4, BmK I6, BmK Ang M1, BmK ITAP3, BmK AGAP, and BmK ITAP. However, the structure-function relationship of these analgesic peptides is far from being elucidated. Our previous studies have identified the importance of W38 for BmK AGAP to maintain its analgesic activity [13]. The two disulfide bonds connecting C22–C46 and C16–C36, respectively, contributed to the analgesic effect of this toxin as well. S54 of BmK9 played a key role in the antinociceptive activity [14], while K8, Y9, N58 in NC domain and Y38, F39, W40 in core domain of BmK AS were closely related to analgesic activity [15]. BmK IT-AP with good analgesic effect had a functional surface constituted of functional site E15 and two binding regions of β-scorpion toxins [16]. Nevertheless, many more function features determining bioactivity of analgesic peptides remain elusive.

Anti-neuroexcitation peptide (ANEP) is a β-anti-excitatory neurotoxin that is isolated from the venom of Chinese *Buthus martensii* Karsch. In addition to the classical four disulfide bridges in the structure of β-toxin, ANEP is a compact of a α-helix and three antiparallel β-strands, and binds to receptor site 4 of sodium channels. The recombinant ANEP that is expressed in our *Escherichia coli* expression system exhibited similar analgesic activity to the native peptide in a mouse-twisting model and the hot plate assay [17]. In order to investigate the binding modes and functional surfaces of ANEP to its target sodium channels, here we generated structural models of the IIS1–S4 domain of mice Na_v_1.7 resting and activated state by using molecular dynamics (MD) simulation. Docking the structural models with ANEP pinpointed that the potential functional residues were responsible for the bioactivity of ANEP. Mutagenesis experiments were in agreement with the prediction results of dynamics simulation, and further in support of the hypothesis that binding orientation is conserved for β-scorpion toxins.

## 2. Results

### 2.1. Molecular Modeling of mNa_v_1.7 (Resting and Activated States)

**Figure 1:**
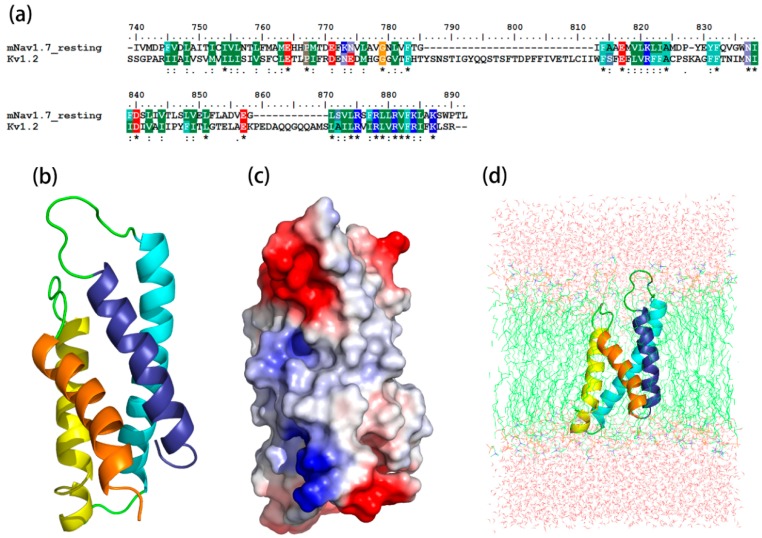
Construction and optimization of the mNa_v_1.7_resting site 4 structure. (**a**) Sequence alignment between mNa_v_1.7_resting and the K_v_1.2_resting; (**b**) The side view of optimized structure by MD simulation; (**c**) A surface potential diagram of optimized structure; (**d**) The optimized structure in the 1-palmitoyl-2-oleoyl-sn-glycero-3-phosphocholine (POPC) membrane.

**Figure 2:**
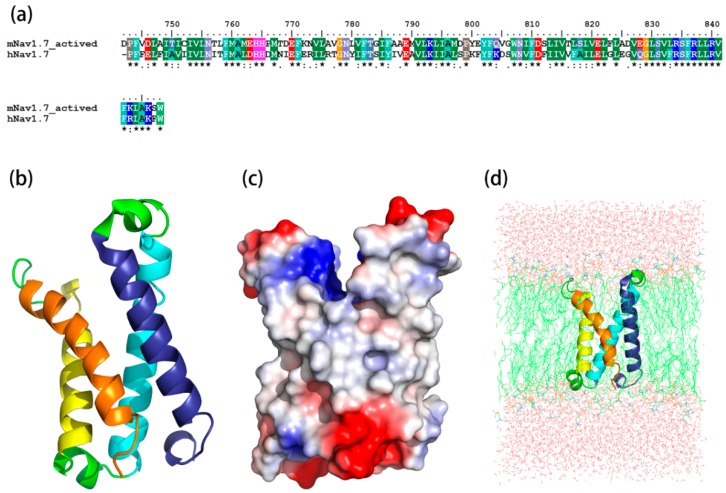
Construction and optimizing of the mNa_v_1.7_activated site4 structure. (**a**) Sequence alignment between the mNa_v_1.7_activated site 4 and the hNa_v_1.7 (PDB ID: 5X0M); (**b**) The side view of optimized structure by MD simulation; (**c**) A surface potential diagram of optimized structure; (**d**) The optimized structure in the POPC membrane.

The sequence identity between the resting state and its template, K_v_1.2 channel, was 45.2%, while the identity between the sequence of the activated state and its template, hNa_v_1.7, was 59%. The most optimized three-dimensional (3D) structures of the mNa_v_1.7 was picked up from 500 models constructed based on probability density functions. Quality evaluation of the chosen model using Ramachandran Plot showed that the Phi/Psi angles of mast residues (~90%) were within the reasonable ranges. To improve the overall quality of the protein model, the structure of homology modeling was energy minimized by molecular mechanics optimization and molecular dynamics simulations in the membrane, and the final stable structures were obtained (Figure 1 and Figure 2).

**Figure 3:**
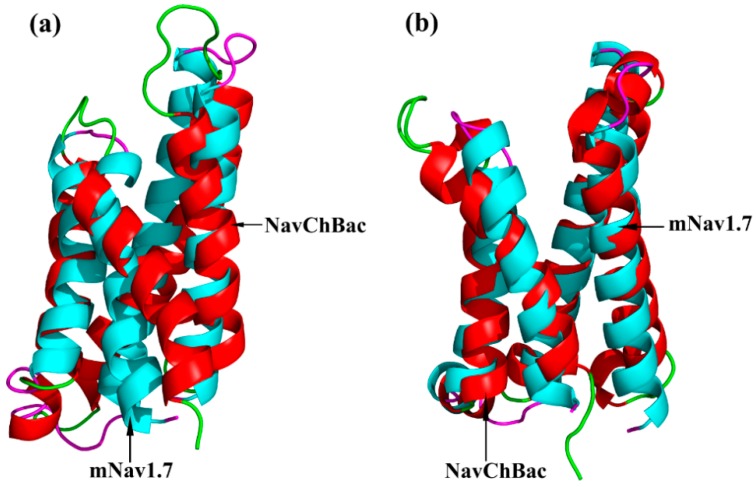
Structure superimposition of mNa_v_1.7 and NaChBac. (**a**) Superimposition of the resting state; (**b**) Superimposition of activated state; mNa_v_1.7 in red with green, NaChBac in blue with pink.

The optimized structures of both states were superimposed with the same states of NaChBac whose conformational rearrangements between resting/closed and activated/open states were extensively studied (Figure 3) [18]. In this study, the overall structure was in accordance with conformational changes of the NaChBac from resting to activated state, which indicated that results of our model construction were reliable. Distinct conformational changes from the resting to the activated state were noticed. The S4 of VSD slid outward through a narrow groove formed by the S1–S3 and tilted sideways at a pivot point that is formed by the middle of the voltage sensor, which resulted in wedge-shaped groove growing wide in the activated state.

### 2.2. Molecular Docking Study

As the ligand, ANEP was docked to the resting and activated states of mNa_v_1.7 by ZDOCK, respectively. Through the ZDOCK, the docking results produced 2000 poses. We selected the three top-scoring (Interactive energy, Van der waals energy, and electrostatic energy) clusters that included the most structures for both resting and activated complexes. The poses from the three clusters with high ZDOCK scores were selected and minimized by RDOCK. To determine which pose was more stable, the interaction energies were calculated, and then the most favorable binding mode was gained. Docking data indicated that in mNa_v_1.7, the residues mainly contributing to bond ANEP were in accordance to the key residues of rNa_v_1.2 with identity 81.3% [19].

### 2.3. Molecular Dynamics Simulations

**Figure 4:**
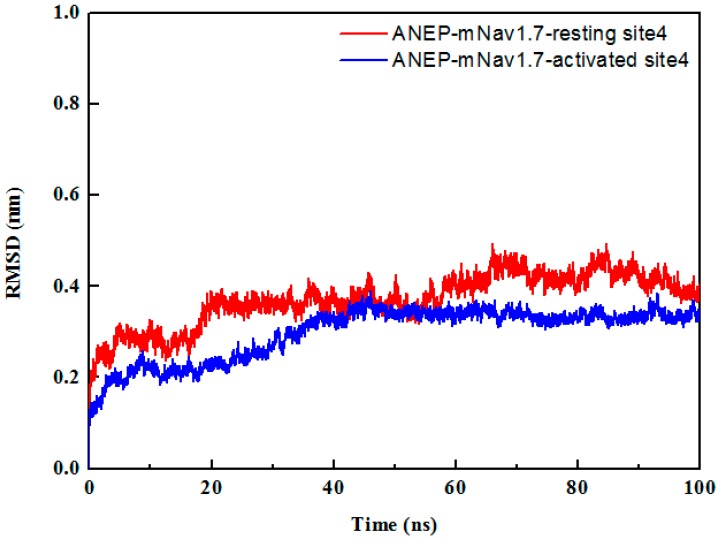
Root mean square deviation (RMSD) of the backbone Cα atoms of the mNa_v_1.7-anti-neuroexcitation peptide (ANEP) complexes of resting and activated states in the 100 ns MD simulation.

To check the stability of the complexes, each docked complex was taken for simulation study for 100 ns in the solvent environment. The equilibration of the systems was monitored through root mean square deviation (RMSD) of the Cα atoms and ANEP-mNa_v_1.7 interaction. According to RMSD from the starting structure, the function of time was analyzed to assess the degree of conformational drift (Figure 4). The RMSD result indicated that the system reached stability after 60 ns of the MD simulation. The lower RMSD values implied that the structures of the complexes were all stable and have no large conformation change in the last 40 ns MD simulations. It further confirmed the feasibility of binding patterns that were predicted by molecular docking. The average structures from the last 40 ns MD simulations were calculated as the final models for ANEP-mNa_v_1.7_resting site 4 and ANEP-mNa_v_1.7_activated site 4 respectively to do the following analysis. 

**Figure 5:**
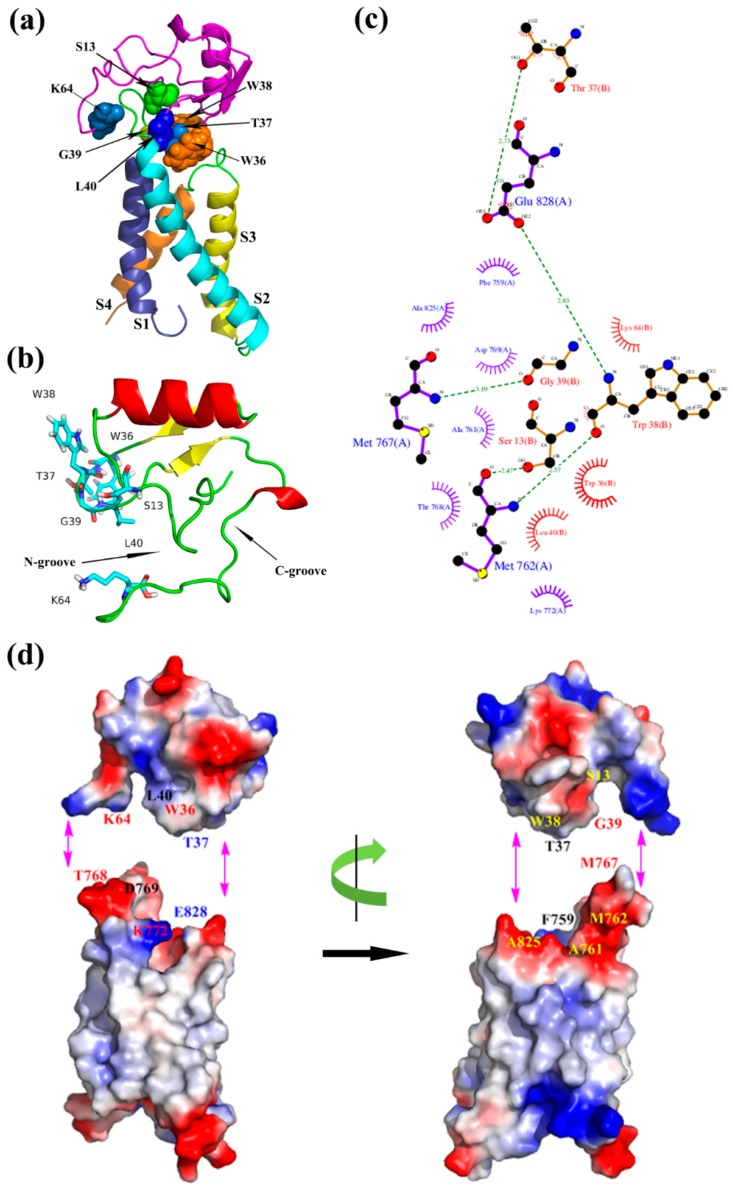
(**a**) Optimized model of ANEP-mNa_v_1.7 site 4 of resting state; (**b**) Binding site of ANEP; (**c**) Interaction between ANEP and mNa_v_1.7-resting site 4 by ligplot; (**d**) Interaction domain of ANEP and mNa_v_1.7-resting site 4. Amino acid residues of interaction are in same color.

By analyzing the binding mode of the resting state (Figure 5), NC-groove was proposed to insert into the wedge space formed by S1–S2 and S3–S4 loop of domain II in mNa_v_1.7, and the close combination of both motifs depended mainly on hydrophobic association and electrostatic interaction. Ser13 at the N-terminus, Lys64 at the C-terminus and the hydrophobic residues Thr37, Trp36, Gly39, and Leu40 of the loop, which connects β2- and β3-strands of ANEP were interacted with S1–S2 loop (K64/T768, W36/K772, L40/D769, S13/A761, S13/M762, G19/M767, T37/F759), whereas Thr37 and Trp38 in β2–β3 loop bound to S3–S4 loop (T37/E828, W38/A825) of receptor site 4, indicating that these amino acids might be essential to the recognition, and thus played important role in and the specificity of the mNa_v_1.7 site4. 

**Figure 6:**
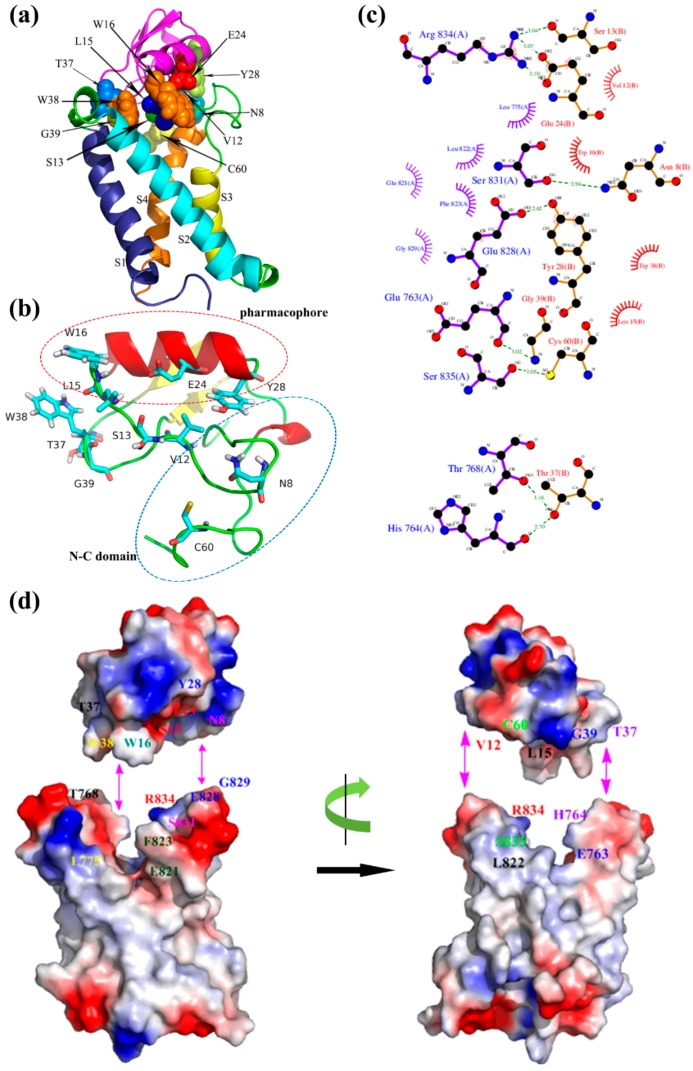
(**a**) Optimized model of ANEP-mNa_v_1.7 of activated state; (**b**) Binding site of ANEP; (**c**) Interaction between ANEP and mNa_v_1.7-activated site 4 by ligplot; (**d**) Interaction domain of ANEP and mNa_v_1.7-activated site 4. Amino acid residues of interaction are in same color.

For the activated binding mode (Figure 6), the key amino acid residues Leu15, Trp16, Glu24, and Tyr28 forming the ‘pharmacophore’ were involved in binding with the S3–S4 loop of mNa_v_1.7 site4 (L15/L822, W16/F823, W16/E821, E24/R834, Y28/E828, Y28/G829) mainly due to hydrophobic association and electrostatic interaction. Also, Asn8 and Cys60 in NC domain, and Val12, Ser13, Trp38, Thr37, and Gly39 in N-groove, primarily bound to S3–S4 loop (N8/S831, C60/S835, V12/R834, S13/R834) and secondarily to the loop of S1–S2 (T37/T768, T37/H764, W38/L775, G39/E763). Val12, Ser13 in the ‘N-groove’ and Glu24 in the ‘pharmacophore’ bound to Arg834 in the mNa_v_1.7 via electrostatic interactions and ionic bond action, respectively. These results were compatible with the study on the interaction between rNa_v_1.2/rNa_v_1.6 and Css4/Cn2 [19,20,21], and were in agreement with previous reports that the ‘pharmacophore’ (E24 and Y28) and N-groove of the scorpion depressant toxin made major contributions to promoting VSD trapping [22]. From the two binding modes, we conjectured that the loop connecting β2- and β3-strands in N-groove, and NC domain played a dual role in receptor site recognition and mode of action.

**Figure 7:**
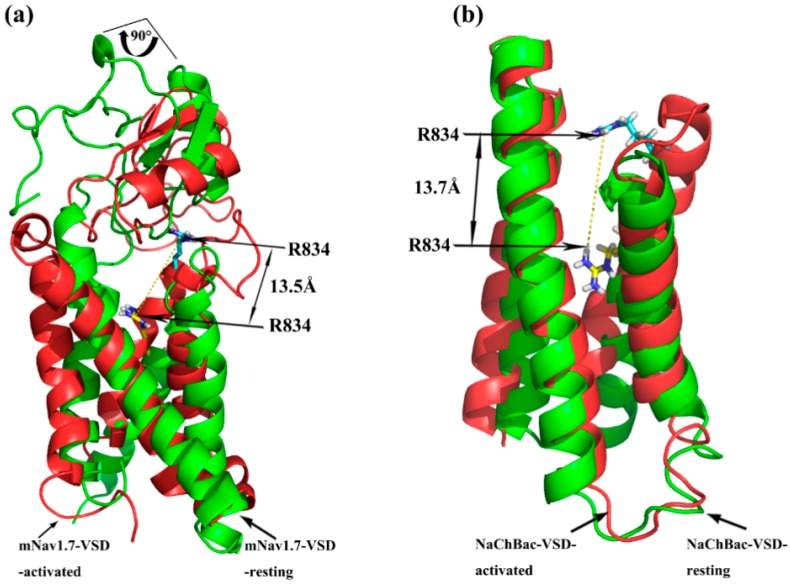
Superimposition of Na_v_-resting and Na_v_-activated model. (**a**) Docking model of mNa_v_1.7 site 4 and ANEP; (**b**) Model of NaChBac-VSD. Na_v_-resting in green, Na_v_-activated in red.

Superimposition of the docking of ANEP to Na_v_1.7-site 4_resting and Na_v_1.7-site 4_activated model was performed for analysis on the trajectory model of Na_v_1.7-site 4. As shown in Figure 7, taking Arg834 in the S3–S4 loop as a reference, it moved outward by ~13.5 Å when the channel was transformed from resting to activated upon binding to ANEP. The same positive charged amino acid residue localized in the NaChBac, also moved out 13.7 Å from resting state to activated status. These results indicated that our simulated conformation altered in a way that is similar to the trajectory model of the NaChBac that Yarov-Yarovoy previously studied [18]. Additionally, ANEP rotated by ~90° around its own axis as IIS4 moved outward, which was consistent with a sequent tilting motion of S3–S4 of voltage-sensing domain (VSD) around S1–S2 as was proposed for a voltage-gated sodium channel from Arcobacter butzleri [23]. According to this widely known hypothesis of voltage-sensor trapping [20,24,25], four conservative positive amino acid residues (R/K) between every two hydrophobic amino acids localized in IIS4 segment play a key role on the function of VSD. By binding to the extracellular end of IIS4, β type scorpion toxin traps this segment in the activated (open) position and further initiated much more negative potentials, eventually leading to enhanced activation. Based on our simulation modeling and superimposition data, we proposed that key amino acid residues in NC domain and β2–β3 loop of ANEP recognized receptor site 4 of resting state mNa_v_1.7 and bound to its extracellular S1–S2 loop, which activated the sodium channel causing the S3–S4 loop of the VSD to move outward. The ‘pharmacophore’ and N-groove of ANEP bound to the newly accessible S3–S4 loop and further trapped it in an outward activated position, in turn enhanced the activation. It is also suggested that the process from resting state to activated state between ANEP and Na_v_1.7 site 4 should be the ‘induced-fit’ model.

### 2.4. Construction, Expression and Purification of Mutants

**Figure 8:**
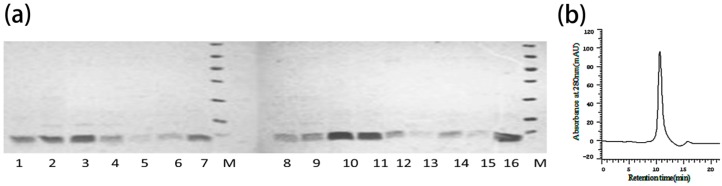
(**a**) 15% SDS-PAGE of the purified mutant proteins. Lane 1, 8, 12: rANEP; Lane 2–7, 9–16: mutants of N8A, V12A, S13A, L15A, W16A, E24A, Y28A, W36A, T37A, W38A, T39A, L40A, C60A, K64⊿; Lane M: Protein Maker; (**b**) RP-HPLC of mutant proteins on an RPC C2/C18 ST column.

Fractions of rANEP and mutants by Chelating Sepharose Fast Flow and the Q-Sepharose Fast Flow chromatography were shown with RP-HPLC to be a single peak (Figure 8b) and produced a single band on reduced SDS-PAGE (Figure 8a). These bands contained a pure peptide, which were identified as rANEP and its mutants.

### 2.5. Analgesic Activities of ANEP Mutants

The mouse-twisting model, one of the classic peripheral pain model, was used to test the analgesic effects of rANEP and mutants, and the results were shown in Table 1.

When compared with the negative control (normal saline), both rANEP and all of the mutant peptides exhibited effective analgesic activity (*n* = 18, *p* < 0.05). When compared with the positive control (rANEP), nine of fourteen sites predicted (N8, L15, E24, W36, T37, W38, T39, C60, and K64) exhibited significant differences in the analgesic activity based on the results of the pharmacological assays (*n* = 18, *p* < 0.05). These sites were mainly distributed in ‘pharmacophore’ domain (L15, E24), N-C binding domain (N8, V12, C60, and K64) and hydrophobic region in β2–β3 loop (W36, T37, W38, T39). The predicted results were generally consistent with the mutation experimental data, and thus we speculated that these sites and their localized motifs were crucial to maintain analgesic activity through the combination with mNa_v_1.7 site 4. On the other side, in the analgesic activities of the mutants S13A, W16A, Y28A, and L40A exhibited not significantly different when compared with those of rANEP (*p* > 0.05). The reason may be that all of the mutant sites were replaced by Ala, which omitted the influence of the site’s electrical properties. Charge properties of some specific sites were crucial to the functional activity of β toxin. Meanwhile, the antinociceptive activity of mutant C60A were significantly decreased than that of the positive control, which might result from the broken disulfide bond in this site, leading to polypeptide conformation change, and thus the decrease of the analgesic effect. 

## 3. Discussion

Here, by using computational modeling and a bioactivity experiment, we identified the binding modes and functional surface of scorpion toxins ANEP to mNa_v_1.7. It is revealed that NC-groove of ANEP was important to the resting state sodium channel receptor recognition, the ‘pharmacophore’ and the N-groove made major contribution to VSD trapping when the channel was activated, and the N-groove, the loop connecting β2- and β3-strands, and NC domain played a dual role in both receptor site recognition and mode of action. Meanwhile, the enlarged N-groove in ANEP mutants exposing more binding space to receptor site accounts for the bioactivity change to some extent (Figure S1). Our results were generally consistent with the functional surfaces of two typical representatives of β-toxin Bj-xtrIT [10] and Css4 [11], which included the conserved putative ‘pharmacophore’, C-terminal region, and the hydrophobic cluster between the β2- and β3-strands of the β-toxin. 

The binding mode of the resting state indicated that Ser13 at the N-terminus, Lys64 at the C-terminus, and the hydrophobic residues Trp38, Thr37, Trp36, Gly39, and Leu40 in the loop between β2- and β3-strands of ANEP were important for receptor site recognition. Site-directed mutagenesis experiments showed that T37A, W38A, and W36A in β2–β3 loop were 83%, 86%, and 114% more active than wild type, respectively, in vivo, suggesting that this hydrophobic cluster might conferred analgesic effect along with binding specificity. Similarly, Tyr40, Tyr42, and Phe44 in the loop between β2- and β3-strands of Css4 [11], Val66, Gln67, Ile68, and 69 of BmK-βIT [16], Val71, Gln72, Ile73, and Ile74 of Bj-xtrIT [10], and Val66, Gln67, Ile68, and Ile69 of BmKIT-AP [26] were also reported to constitute the receptor site recognition function surface of toxins and contribute to the toxin effects as well. The simulation structures of ANEP and its mutants with the increasing analgesic activity showed that the mutations of amino acids might have broke the interactions within the NC domain that existed in the wild type of toxin (Figure S1), which moved the loop of β2–β3 up, made the side chains of W36 and W38 extend inward, and thus enlarged the N-groove. When W36, T37, or W38 were mutated to alanine, the side chains also downsized, allowing for more space to expose the enlarged N-groove, which hence further fortified the antinociceptive effect of ANEP (Figures S1 and S2).

Pharmacophore, composed mainly of a cluster of putative conserved amino acids in α-helix and its vicinity, is a common function domain to all β-toxins. The result of MD simulations identified that Ser13, Leu15, Trp16, Glu24, and Tyr28 in the ‘pharmacophore’ of ANEP bound to the L822, F823, E821, R834, E828, and G829 at S3–S4 loop and the extracellular region of IIS4, the VSD domain of mNa_v_1.7 activated state. E24 is the center of the hot spot to this conserved ‘pharmacophore’, and thus is particularly essential to VSD trapping. In our activated state model of ANEP-mNa_v_1.7, E24 first interacted with the newly moving-outward R834, and the later was also involved in the interaction with Y28, another highly conserved amino acid in the ‘pharmacophore’ of β-toxin. When Glu24 was mutated to alanine, interaction E24-R834 lost their charge association, making D19 side chain extend into the wedge space formed by S1–S2 and S3–S4 loops, where this negative charge amino acid met and interacted with R837. Meanwhile, the released R834 was interacted with S13 (Figure S2). The central role of glutamic acid on the toxin ‘pharmacophore’ has been demonstrated by multiple authors [10,11]. Cohen et al. reported that Glu28 of Css4 constituted the hot spot in the interacting surface with ion channel, while side chains of Tyr24 and Gln32 projected to the solvent and flank Glu28. Resembling both chemically and functionally, Glu30 of Bj-xtrIT formed the hot spot interacting with the putative charge on the receptor, whereas Tyr26 and Val34 functioned as a seal to occlude bulk solvent from the high-energy point of interaction [11]. Our mutant toxin E24A exhibited a more significant analgesic effect than the wild type polypeptide. Interestingly, however, mutant of another highly conserved amino acid, Y28A, did not show much different antinociceptive activity when compared with rANEP. Y28 side chain played crucial role on keeping the active function surface by projecting to the flank key amino acids in ‘pharmacophore’. We assumed that the charge change of the side chains accounts for these unexpected results, which need to be further investigated experimentally. 

By binding to the positive R834 on the gating surface of receptor site 4, Ser13 in the N-groove was not only involved in receptor site recognition, its negative charge side chain also trapped the moving-outward R834 at the VSD of mNa_v_1.7 in the open/activated state. However, in our bioassay, the antinociceptive effect of mutant S13A was not significantly different from its wild type toxin either. Nevertheless, when Ser13 was replaced by arginine, another amino acid with same positive charge side chain, this mutant toxin S13R exhibited a much different analgesic effect when compared with rANEP (unreported data). Evidently, charge properties of Ser13 side chains were crucial to the functional activity of the toxin. 

NC-domain played dual role both on site recognition and toxin action. As our docking models showed (Figure S1), in wild type ANEP, K64 at C-terminus interacted with K11 of N-terminus to stabilize NC-domain stable. Also, the K64 was found to form an in-upward hook. After mutation (K64⊿), the interaction between N- and C-terminus changed from K64-K11 to T59-G9, and thus enlarged N-groove generating more surface area bound to receptor site 4. Likewise, Trp58 of Css4 and Trp54 of Ts1 [27] have been reported to determine the toxins specificity and binding affinity to their receptor sites. Our mutation bioassay also showed that mutants C60A and K64A exhibited significantly different pharmaceutical effects than their wild type polypeptide. In addition, the substitution of Asn8 in the N-terminus also altered the mutant toxin analgesic activity evidently, suggesting that N-terminus might get involved in toxin function as well.

Based on the above analysis, we believe that this study could provide some theoretical support for the modification and design of analgesic peptides that target Na_v_1.7. However, intensive study should be made in order to obtain bioactive peptides with a better curative effect and less side effects. For example, the amino acid residues in important binding regions need to be mutated to some other residues instead of just alanine (e.g., change the charge or polarity of these residues) so that we could have a further understanding of the interactions of these residues. Moreover, some unimportant sites and domains can be deleted to reduce the volume of the peptides. In addition, the selectivity of the peptides for other sodium channels isoforms related to side effects, such as Na_v_1.4 (mainly expresses in muscle) and Na_v_1.5 (mainly expresses in cardiac), is necessary to be considered for the design of analgesic peptides to avoid the sake of safe treatment.

## 4. Conclusions

Theoretically and experimentally, we addressed the function surfaces of ANEP, a depressant scorpion toxin that binds to VGSCs receptor site 4. A ‘pharmacophore’ reserved to various toxins and the loop between β2- and β3-strands contribute to toxin analgesic activity mainly. The newly identified N-groove and NC-domain were important to the polypeptide bioactivity by stabilizing the function surface structure. 

## 5. Materials and Methods

### 5.1. Homology Models

The homology modeling software MODELLER9.9 was applied to construct the model of the isolated IIS1–S4 domains of mice Na_v_1.7 (resting and activated state) and ANEP [28]. The optimized structure of ANEP was obtained, as described previously [29]. The sequence of the IIS1–S4 was downloaded from Uniprot database (Q62205) to generate the structure of the IIS1–S4 domains of mice Na_v_1.7. In order to obtain the model of the resting and activated state, we used the constructed structural model of the resting state of the Kv1.2 channel [30] and the crystal structures of the human Na_v_1.7 (hNa_v_1.7) (PDB ID: 5X0M) [1] as a template, respectively. The quality of the model was further assessed with Ramachandran plot.

### 5.2. IIS1–S4 Domains Molecular Dynamics Simulations

The simulations were performed using the GROMACS 5.1.1 package [31] and the topology file of the proteins were generated using GROMOS96-53a6 force field [32]. For both resting and activated states, the pore region was embedded in a POPC (1-palmitoyl-2-oleoyl-sn-glycero-3-phosphocholine) bilayer using the INFLATHGRO tools [33] and the protein complex was inserted into a water box that used the SPC water model. The solvated system was neutralized by adding suitable number of Na+ and Cl− ions to maintain electro-neutrality of the system. The energy was minimized using the steepest descent algorithm, followed by positional restrained MD, which includes a 100 ps isochoric-isothermal (NVT) simulation to 300 K and a 1 ns isothermal-isobaric (NPT) simulation to 1 atm. The temperature and pressure was maintained using the Berendsen thermostat temperature coupling and Parrinello-Rahman pressoatat method [34], respectively. The long-range electrostatic interactions were computed using Particle Mesh Ewald summation method [35]. The MD simulations used a timestep of 2 fs with bond distances being constrained by the LINCS algorithm [36] and water geometries being constrained by SETTLE [37]. The Cα of the peptide were restrained to their initial position with a force constant of 1000 kJ/(mol nm^2^). Finally, a 100 ns molecular dynamics simulation was carried out.

### 5.3. Molecular Docking

The complexes of the ANEP and mNa_v_1.7 were generated by molecular docking calculations, which were used as the starting configuration of subsequent unbiased MD simulations. Prior to docking, the ANEP and mNa_v_1.7 were converted to CHARMm atom types as required by the docking program ZDOCK, a docking algorithm for protein-protein complexes. We used the ZDOCK program in the Accelrys Discovery Studio 3.0 (DS3.0) to dock the ANEP to the isolated IIS1–S4 domain of mNa_v_1.7, and 2000 poses were generated for toxin-channel pair. The most plausible poses were selected according to experimental data as well as docking scores. RDOCK, an energy minimization algorithm for refining ZDOCK results, was used to optimize the poses by default settings, which was a CHARMm force field based refinement algorithm that performs limited molecular dynamics to fine-tune protein–protein complexes from ZDOCK. RDOCK used a two-stage scoring function: van der Waals energy was first calculated to discard docking poses with clashes and then the poses were scored based on desolvation and electrostatic energies. Lastly, DS3.0 was used as an interface to ZDOCK and RDOCK and to visualize the result.

### 5.4. The Complex Molecular Dynamics Simulations

All of the molecular dynamics simulations were preformed using GROMACS 5.1.1 package [31] at 1 atm and 310 K, with periodic boundary condition and a 2-fs time step. The GROMOS96-53A6 force field was used to describe the interatomic interactions in lipids, proteins, and ions. The SPC water model was used to describe water molecules. All of the bond lengths were constrained using the LINCS [36] algorithm. Finally, some 100 ns molecular dynamics simulations were carried out. The simulations trajectories were analyzed using tools from the GROMACS package and were visualized using PyMOL and VMD molecular visualizer software. 

### 5.5. Strains, Materials, and Animals

Plasmid pSYPU-1b-Tag_(his)_-ANEP [17], *E. coli* strains DH5α and BL21 (λDE3) were stored in our laboratory. Restriction endonucleases, Taq DNA polymerase and T4 DNA ligase were purchased from TaKaRa (Dalian, China. The primers were synthesized by JINSITE (Nanjing, China). Chelating Sepharose Fast Flow and SP (sulfopropyl) Sepharose fast flow were purchased from GE Healthcare (Pittsburgh, PA, USA). The mice used for the analgesic activity bioassay were Kunming mice (SPF) from the Animal Center of Shenyang Pharmaceutical University. All of the animal protocols were approved by the Institutional Animal Care and Use Committee.

### 5.6. Site-Directed Mutagenesis of ANEP

Based on the amino acid sequence of BmK ANEP, the mutagenic primers used to generate the desired mutations were designed (Table 2). Using pSYPU-1b-Tag_(his)_-ANEP as a template, some mutants of ANEP were created by three-step PCR. The first PCR products were generated by the general sense primer ANEP-F and the antisense primer of each specific mutant, while the second step were introduced by the mutant sense primer and ANEP-R. After being purified by DNA Gel Extraction Kit, the first and second PCR products were amplified by the pair of general primers, ANEP-F and ANEP-R, using an overlapping extension method. All of the target genes were inserted into plasmid pSYPU-1b, as described to obtain *E. coli* BL21 (λDE3) cells harboring various recombinant pSYPU-1b plasmids [17]. All clones were screened and selected by sequencing the entire PCR fragments.

### 5.7. Expression and Purification of ANEP and Its Mutants

The expression and purification procedure of rANEP and its mutants were performed according to the previous report [17]. The purity of the sample was checked on 15% SDS-PAGE and RP-HPLC. The protein concentration was determined by the Bradford method with bovine serum albumin as a standard.

### 5.8. Analgesic Activity Assays

The mouse-writhing test was carried out as described by Fennessy and Lee. To perform the bioassay, rANEP and its mutants were dissolved in normal saline at a concentration of 0.15 mg/mL, and the toxin solution (0.2 mL per 20 g body weight) was injected intravenously into the tails of the mice (male and female, 18–20 g body weight; *n* = 18), using normal saline as a negative control. Twenty minutes later, 0.2 mL 0.6% acetic acid solution was then injected intraperitoneally. Five minutes later, the number of writhes was counted within a 10-min period. The results were analyzed using a Statistical Package for the Social Science. Statistical analysis of the data was provided as mean ± SEM. Group statistical significance was assessed using one-way ANOVA, followed by a Dunnett’s test.

## Figures and Tables

**Table 1:** Overview of expression and analgesic activity of rANEP and its mutants (*n* = 18).

Group 1	Dosage (mg/kg)	Number of Writhes (Mean ± SEM)	Inhibition Efficiency (%) ^1^	Relative Activity (%)
NS	-	47.67 ± 1.77	-	-
rANEP	1.5	29.00 ± 3.41 *	39.17%	100
rANEP-N8A	1.5	21.60 ± 3.7 *^,^^2^	54.68%	132
rANEP-V12A	1.5	20.33 ± 2.98 *^,^^2^	57.35%	143
rANEP-S13A	1.5	29.70 ± 2.5 *	37.69%	96
rANEP-L15A	1.5	22.00 ± 1.25 *^,^^2^	53.85%	132
rANEP-W16A	1.5	23.88 ± 1.55 *	49.90%	121
rANEP-E24A	1.5	20.50 ± 3.26 *^,^^2^	57.00%	141
rANEP-Y28A	1.5	24.75 ± 2.6 *	48.06%	114
rANEP-W36A	1.5	13.56 ± 2.36 *^,^^2^	71.55%	214
rANEP-T37A	1.5	15.89 ± 3.11 *^,^^2^	66.67%	183
rANEP-W38A	1.5	15.56 ± 2.47 *^,^^2^	67.36%	186
rANEP-G39A	1.5	21.93 ± 2.1 *^,^^2^	55.12%	132
rANEP-L40A	1.5	30.33 ± 2.8 *	35.71%	93
rANEP-C60A	1.5	35.21 ± 2.1 *^,^^2^	26.14%	67
rANEP-K64⊿	1.5	14.98 ± 3.16 *^,^^2^	68.58%	175

^1^ The inhibition efficiency is the ratio (*T*_0_ − *T*)/*T*_0_, where *T*_0_ is the mean number of writhes in the negative control group and *T* is the mean number of writhes in the experiment groups with rANEP and mutants (*n* = 18); ^2^ *p* < 0.05 vs. rANEP; * *p* < 0.05 vs. Normal Saline.

**Table 2:** Primers used to construct different mutations of ANEP.

Name	Nucleotide Sequence (5′-3′)	Orientation
ANEP-F	CATGCCATGGGACATCATCATCATCATCACGATGGATATATAAGAGGAAGTAACGGATG	Sense
ANEP-R	CGGGATCCTTACTTTTTGCCACCGCATGTATTACTTTCA	Antisense
N8A	CATGCCATGGGACACCACCACCACCACCACGATGGATATATAAGAGGAAGT**GCG**GGATG	Sense
V12A	AACGGATGCAAG**GCG**TCATGC	Sense
	ATGA**CGC**TTGCATCCGTTACT	Antisense
S13A	CAAGGTT**GCG**TGCTTATGGGGA	Sense
	CATAAGCA**CGC**AACCTTGCATCC	Antisense
L15A	TCATG**CGC**GTGGGGAAATG	Sense
	TCCCCA**CGC**GCATGAAACCTT	Antisense
W16A	TCATGCTTA**GCG**GGAAATGAC	Sense
	GACATTTCC**CGC**TAAGCATGAA	Antisense
E24A	CAATAAA**GCG**TGCAGAGC	Sense
	TGCA**CGC**TTTATTGCAACCGTCATT	Antisense
Y28A	AGAGCG**GCG**GGTGCCTCTT	Sense
	GAGGCACC**CGC**CGCTCTG	Antisense
W36A	TATGGTTATTGC**GCG**ACCTGGGGA	Sense
	AAGTCCCCAGGT**CGC**GCAATAACCATAAGA	Antisense
T37A	TGCTGG**GCG**TGGGGACT	Sense
	TCCCCA**CGC**CCAGCAATAA	Antisense
W38A	ACC**GCG**GGACTTGCATGC	Sense
	TCC**CGCG**GTCCAGCAATAAC	Antisense
G39A	ACCTGG**GCG**CTTGCATGCTG	sense
	CAGCATGCAAG**CGC**CCAGGT	Antisense
L40A	TGGGGA**GCG**GCATGCTG	Sense
	CAGCATGC**CGC**TCCCCA	Antisense
C60A	CGGGATCCTTACTTTTTGCCACCCGCTGTATTACT	Antisense
K64⊿	CGGGATCCTTATTTGCCACCGCATGTATTACTT	Antisense

The underlined CCATGG and GGATCC represent restriction enzyme site *Nco* I and *BamH*I respectively.

## Supplementary Materials

The following are available online at www.mdpi.com/2072-6651/9/12/387/s1, Figure S1: Structure of the ANEP and mutants by the homology modeling; Figure S2 Interaction model of ANEP mutants and mNav1.7_activated site 4.
