# Supplementary material for: Investigation of Binding Modes and Functional Surface of Scorpion Toxins ANEP to Sodium Channels 1.7
Source: Toxins (Basel). 2017 Nov 29;9(12):387. doi: 10.3390/toxins9120387 (PMC5744107; doi:10.3390/toxins9120387)
Supplement: Supplementary file 1 [file toxins-09-00387-s001.pdf]

Supplementary Materials: Investigation of Binding Modes and Functional Surface of Scorpion Toxins ANEP to Sodium Channels 1.7

Yongbo Song, Zeyu Liu, Qi Zhang, Chunming Li, Wei Jin, Lili Liu, Jianye Zhang and Jinghai Zhang

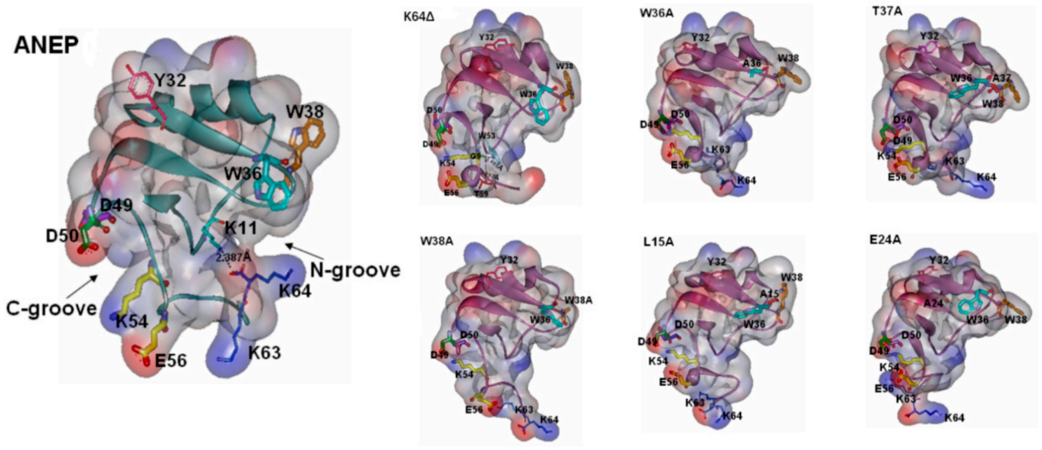

Figure S1 Structure of the ANEP and mutants by the homology modeling

We optimized mutants with significantly increased analgesic activity by the MD simulation, and analyzed the average structure of our selected stabilized structure. Our results showed that the enlarged N-groove was closely related to the improved analgesic activity and the binding mode of NC-domain played an important role as well.

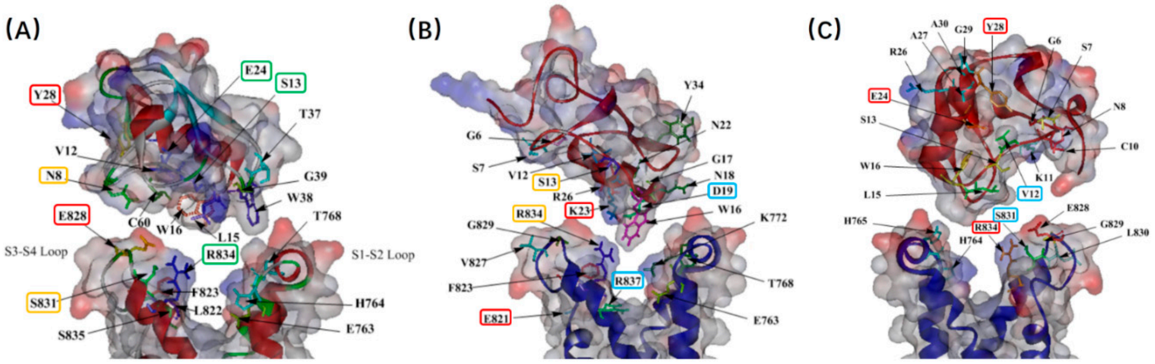

Figure S2 Interaction model of ANEP mutants and mNav1.7_activated site 4. (a) Interaction model between ANEP and mNav1.7_activated site 4; (b) Interaction model between E24A and mNav1.7_activated site 4; (c) Interaction model between K64⊿ and mNav1.7_activated site 4. Amino acid residues of interaction are in the same color.

The mutants with significant increased analgesic activity were carried out docking with the mNa$_V$1.7 activated site 4, and then two typical docking models were obtained and optimized in the POPC membrane by MD simulations. And the average structures after the MD were analyzed (Figure S2).

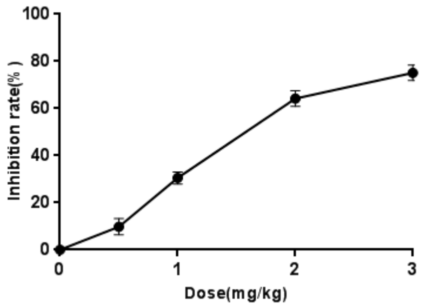

Figure S3 Dose-effect curve for rANEP plotted according to acetic-acid-induced writhing response in mice.

As shown in Figure S3, there was a dose-dependent relationship between rANEP and inhibition rate in the mouse-writhing model. Based on this result, the choice of dose is 1.5mg/kg (Table1), slightly less than IC_{50} in the analgesic activities assays.
